# Heterologous expression and characterization of polyhydroxyalkanoate synthase genes from haloarchaeal strains in *Haloferax mediterranei*

**DOI:** 10.3389/fmicb.2026.1754904

**Published:** 2026-03-18

**Authors:** Keisuke Wada, Kazunori Ushimaru, Shun Sato, Tokuma Fukuoka

**Affiliations:** Research Institute for Sustainable Chemistry (RISC), National Institute of Advanced Industrial Science and Technology (AIST), Tsukuba, Ibaraki, Japan

**Keywords:** *Halalkalicoccus jeotgali*, haloarchaea, *Haloferax mediterranei*, *Natronococcus occultus*, PHA synthase, *phaEC*, poly(3-hydroxybutyrate-*co*-3-hydroxyvalerate), polyhydroxyalkanoate

## Abstract

**Introduction:**

Polyhydroxyalkanoates (PHAs) are promising materials for building a sustainable society due to their excellent biodegradability. Some haloarchaea, which require high salt concentrations for growth, possess class III PHA synthases (PhaECs) and produce poly(3-hydroxybutyrate-*co*-3-hydroxyvalerate) (PHBV). Since PhaECs are a major factor in determining monomer compositions and molecular weights that affect the physical properties of PHAs, understanding their functionalities is essential to widen their applications. This study aimed to systematically evaluate the functionality of haloarchaeal *phaEC*s under uniform conditions using a heterologous expression system in a PHA-negative mutant of *Haloferax mediterranei*.

**Methods:**

To investigate the characteristics of *phaEC*s from the five strains of haloarchaea, the genes were introduced into the strain of *H. mediterranei* lacking the original *phaEC*. The recombinant strains were subjected to PHA production evaluation.

**Results:**

All transformants produced PHBV, whereas some native strains did not, indicating host-dependent limitation. The molecular weights of PHBVs produced by the strains possessing *phaEC*s from *Halalkalicoccus jeotgali* and *Natronococcus occultus* were higher than those of PHBVs produced by *H. mediterranei*. In the presence of propionate, a precursor of 3-hydroxyvalerate (3HV), a strain containing *phaEC* derived from *H. jeotgali* boosted the 3HV composition in PHBV without significantly suppressing cell growth or decreasing molecular weight due to propionate toxicity.

**Discussion:**

These results demonstrated that *phaEC*s from haloarchaeal strains other than *H. mediterranei* also has the potential to produce high-molecular-weight-PHBV. Notably, *phaEC* from *H. jeotgali* exhibited exceptional potential for producing PHBV with both high molecular weight and enhanced 3HV composition. This work establishes a systematic framework for functional genomics of haloarchaeal PHA synthases and provides insight for engineering tailor-made bioplastics using extremophiles.

## 1 Introduction

Haloarchaea are a type of archaea that require high concentrations of salts for their activities and stabilities. Haloarchaeal strains have some special features that allow them to survive harsh conditions. For example, haloarchaeal enzymes containing a high proportion of acidic amino acids tend to be stable at high salt concentrations (Siroosi et al., 2014). Some haloarchaeal strains have light-driven membrane enzymes called rhodopsins, which pump ions, including protons and chlorides. Proton-pumping rhodopsins exhibit considerable potential for use in metabolic engineering (Toya et al., 2022) and various industrial processes (Singh et al., 2021). Most haloarchaeal strains produce antioxidant pigments such as *β*-carotenes, lycopene, phytoene, and bacterioruberins (Grivard et al., 2022). Additionally, some haloarchaeal strains produce polysaccharides, including anionic sulfated heteropolysaccharides (López-Ortega et al., 2024) and inulin (Aragón-León et al., 2023). Notably, some haloarchaeal strains produce polyhydroxyalkanoates (PHAs) (Han et al., 2010a), which exhibit excellent thermoplasticity and biodegradability in natural environments (Suzuki et al., 2021). These characteristics have attracted considerable attention in industrial biotechnology (Chen and Jiang, 2018).

PHAs are microbial polyesters that can be used as thermoplastics. Given their excellent biodegradability, the growing application of PHAs to address global concerns regarding plastic waste in natural environments drives advances in the production and structural diversity of PHA. PHA producers include not only bacteria, but also some archaea, including haloarchaea. Previous studies have shown that various haloarchaeal strains can produce poly(3-hydroxybutyrate-*co*-3-hydroxyvalerate) (PHBV) with varying productivities and 3-hydroxyvalerate (3HV) composition, depending on the strain (Han et al., 2010a). These strains have been investigated to elucidate the genetic organization of PHA biosynthetic genes, and some genomes have also been sequenced. Genetic analyses revealed the presence of the PHA synthase gene, which encodes the key enzyme for PHA biosynthesis, in PHA-producing haloarchaea. The genes consist of two open reading frames encoding PhaC and PhaE heterosubunits, which assemble into Class III PHA synthase (Wang et al., 2019; Vuong et al., 2021). These studies suggest that PHA production in haloarchaea depends on PHA synthase genes, and a wide distribution of PHA-producing ability in haloarchaea may promote diversity in PHA productivity and structure. However, these factors also reflect host-derived influences, such as the supply of monomer components and the intracellular capacity for PHA accumulation. Hence, evaluation of each PHA synthase gene in haloarchaea requires a uniform heterologous expression system. *Escherichia coli* and *Saccharomyces cerevisiae* are experimental hosts for the heterologous expression of target genes to assess their functions. Numerous bacterial PHA synthase genes have been investigated using *E. coli* as an expression host (Agus et al., 2006; Hiroe et al., 2012; Mishra et al., 2025). However, enzymes from haloarchaea, which are adapted to high-salinity conditions, function poorly in a low salt concentration environment inside *E. coli* cells (Amoozegar et al., 2017). This has led to a lack of evaluation results for PHA synthase genes in haloarchaea. Therefore, a systematic framework for evaluating PHA synthase genes in haloarchaea needs to be developed.

*Haloferax mediterranei*, a haloarchaeon that requires a minimum of 1 M salt for growth (Oren and Hallsworth, 2014), has a salt requirement that is lower than required by most other haloarchaea. Also, *H. mediterranei* is a well-known PHA producer capable of producing PHBV from unrelated carbon sources. Our previous study demonstrated that *H. mediterranei* produced PHBV with 7 mol% 3HV, and the PHBV had a weight-average molecular weight (*M*_w_) of 4.4 × 10^6^ g/mol (Ino et al., 2020). A cold-drawn film made from the PHBV had a tensile strength of 259 MPa and Young’s modulus of 0.9 GPa, comparable to those of a film made from ultrahigh-molecular-weight (UHMW) poly(3-hydroxybutyrate) (Iwata et al., 2003). We further determined the *M*_w_ of the PHBV produced by *H. mediterranei* could be increased to 5.8 × 10^6^ g/mol in nutrient-limiting medium (Sato et al., 2021). These results suggest that *H. mediterranei* possess a sufficient monomer supply to produce PHAs. A recombinant system for *H. mediterranei* has been developed and is suitable for evaluating PHA synthase genes in haloarchaea. Moreover, the fermentation process of *H. mediterranei* can be simplified by exploiting their unique properties. The risk of contamination remains low until the end of the culture, even when using non-sterile instruments. This is because *H. mediterranei* grows in media containing high salt concentrations, where other microbes cannot grow. *H. mediterranei* cells lyse in hypotonic water due to changes in osmotic pressure, thus simplifying the extraction processes of intracellular products. Therefore, *H. mediterranei* is a promising host for next-generation microbial factories.

Recent studies have increasingly revealed that PHA biosynthesis in *H. mediterranei* is regulated and executed in a more complex manner than previously assumed. Transcriptomic analyses demonstrated that the three *phaC* paralogs, once regarded as cryptic, are transcriptionally active and exhibit growth phase-dependent expression (Vanden Haute et al., 2026). This finding indicates that PHA production in *H. mediterranei* involves coordinated and hynamic regulation at the gene expression level. In addition, biochemical characterization of the native PhaC has shown pronounced haloalkaliphilic and thermostable properties, which may influence polymer chain elongation and overall PHA accumulation *in vivo* (Alsafadi et al., 2025). Furthermore, metabolic engineering studies revealed that homologous overexpression of phaEC enhances PHBV productivity and increase the 3HV composition, underscoring the importance of enzyme abundance as well as inherent catalytic properties in determining polymer composition (Simica et al., 2025). Taken together, these studies suggest that both enzyme-dependent factors and host physiological characteristics contribute to PHA biosynthesis in *H. mediterranei*, highlighting the need for a systematic and uniform framework to evaluate haloarchaeal PHA synthases.

In this study, we investigated the functional diversity of haloarchaeal *phaEC* using a heterologous expression system based on a PHA-negative mutant of *H. mediterranei*. We hypothesized that sequence variations in haloarchaeal *PhaEC*s lead to differences in PHA biosynthesis, particularly in monomer composition and molecular weight. To evaluate these differences under uniform conditions, we developed a systematic *in vivo* evaluation framework using the haloarchaeal vector pWL102 and *H. mediterranei* as the expression host, enabling direct comparison of *phaEC*s from five haloarchaeal strains. Sequence analysis identified several strains with *phaEC*s potentially suitable for PHBV production, and all transformants expressing haloarchaeal *phaEC* produced PHBV, despite the ability to produce PHBV in the native host strains. These results reveal not only PHA synthase gene-dependent variations in PHBV structures but also host-dependent factors in PHA biosynthesis, demonstrating the usefulness of this framework for functional genomics of haloarchaeal PHA synthases. Overall, this work provides a basis for developing tailor-made bioplastics and advancing extremophile-based industrial biotechnology.

## 2 Materials and methods

### 2.1 Phylogenetic analysis

All haloarchaeal strains used in this study are summarized in Supplementary Table S1. *Halogranum salarium* NBRC110682 was renamed *H. rubrum* (Tan et al., 2024). PhaC sequences of all haloarchaeal strains and *Allochromatium vinosum* DSM180 strain, which was set as the outgroup, were obtained from NCBI GenBank. Although *H. mediterranei* harbors three types of *phaC*, the enzyme considered to work as the main contributor to PHA synthesis in *H. mediterranei* (Han et al., 2010b) was employed among them. Multiple sequence alignments were performed using the MUSCLE (Edgar, 2004), and phylogenetic analyses were conducted using the neighbor-joining methods with the Poisson substitution model for amino acid sequence in the MEGA11 software (Tamura et al., 2021) with default parameters. The robustness of the tree topology was evaluated by bootstrap analysis with 1,000 replicates.

### 2.2 Microbial strains and cloning

All strains and plasmids used in this study were summarized in Table 1. *Haloarcula hispanica* NBRC102182 (≡ Y27^T^), *Halalkalicoccus jeotgali* NBRC110681 (≡ B3^T^), *H. mediterranei* NBRC14739 (≡ R-4^T^), *H. rubrum* NBRC110682 (≡ B-1^T^), and *Natronococcus occultus* NBRC102186 (≡ SP4^T^) were purchased from the Biological Resource Center, National Institute of Technology and Evaluation (NITE) (NBRC, Tokyo, Japan). These strains were reconstituted with the respective rehydration fluids recommended by the NBRC. Cells were harvested by centrifugation, and genomic DNA was extracted using NucleoSpin Tissue (Takara Bio, Shiga, Japan) according to the manufacturer’s instructions. These DNAs were used as templates to generate the fragment of *phaRP* promoter derived from *H. mediterranei* and *phaEC*s from each haloarchaeon.

**Table 1:** Strains and plasmids used in this study.

Strain or plasmids	Relevant characteristics	References
Strain		
*Haloferax mediterranei* NBRC14739	Wild-type strain ≡ R-4^T^	Rodriguez-Valera et al. (1983)
*Haloarcula hispanica* NBRC102182	Wild-type strain ≡ Y27^T^	Juez et al. (1986)
*Natronococcus occultus* NBRC102186	Wild-type strain ≡ SP4^T^	Tindall et al. (1984)
*Halalkalicoccus jeotgali* NBRC110681	Wild-type strain ≡ B3^T^	Roh et al. (2007)
*Halogranum rubrum* NBRC110682	Wild-type strain ≡ B-1^T^	Kim et al. (2011)
DEC	Δ*phaEC* mutant of *H. mediterranei*	Lu et al. (2008)
DEC-null	DEC [pWL102–P*_phaRP_*]	This study
DEC-*phaEC(Hme)*	DEC [pWL102–P*_phaRP_*–*phaEC(Hme)*]	This study
DEC-*phaEC(Hhi)*	DEC [pWL102–P*_phaRP_*–*phaEC(Hhi)*]	This study
DEC-*phaEC(Noc)*	DEC [pWL102–P*_phaRP_*–*phaEC(Noc)*]	This study
DEC-*phaEC(Hje)*	DEC [pWL102–P*_phaRP_*–*phaEC(Hje)*]	This study
DEC-*phaEC(Hru)*	DEC [pWL102–P*_phaRP_*–*phaEC(Hru)*]	This study
Plasmids		
pWL102	10.5 kbp; shuttle vector; Amp^R^ Pra^R^	ATCC77216
pWL102–P*_phaRP_*	10.5 kbp; *phaRP* promoter from *H. mediterranei*	This study
pWL102–P*_phaRP_*–*phaEC(Hme)*	12.5 kbp; *phaEC* from *H. mediterranei* under *phaRP* promoter	This study
pWL102–P*_phaRP_*–*phaEC(Hhi)*	12.4 kbp; *phaEC* from *H. hispanica* under *phaRP* promoter	This study
pWL102–P*_phaRP_*–*phaEC(Noc)*	12.4 kbp; *phaEC* from *N. occultus* under *phaRP* promoter	This study
pWL102–P*_phaRP_*–*phaEC(Hje)*	12.5 kbp; *phaEC* from *H. jeotgali* under *phaRP* promoter	This study
pWL102–P*_phaRP_*–*phaEC(Hru)*	12.5 kbp; *phaEC* from *H. rubrum* under *phaRP* promoter	This study

Square brackets denote plasmid carrier state.

*Escherichia coli* DH5α high champion (SMOBIO Technology, Hsinchu, Taiwan) was used for DNA manipulation. Plasmid pWL102 was used as the parental vector (Lam and Doolittle, 1989). Ampicillin sodium (100 mg/L), pravastatin sodium (4.5 mg/L) (Ozawa et al., 2005) and agar (15 g/L) were added as necessary. The *phaEC*-deficient strain of *H. mediterranei* (DEC) was constructed using the same methods as in a previous study (Lu et al., 2008). The primers and product sizes were summarized in Supplementary Table S2. DNA fragments containing *phaRP* promoter from *H. mediterranei* (P*_phaRP_*) (Cai et al., 2015) was amplified using the appropriate primers and PrimeSTAR Max DNA polymerase (Takara Bio) and subcloned into *Xba*I sites in pWL102 using In-Fusion HD Cloning Kit (Takara Bio), resulting in pWL102-P*_phaRP_*. The P*_phaRP_* fragment contained an *Nde*I site immediately above the start codon. DNA fragments containing *phaEC*s derived from each strain were also amplified with the genomic DNA as templates using the primers and PrimeSTAR Max DNA polymerase (Takara Bio). These fragments were subcloned into *Nde*I and *Kpn*I sites of pWL102-P*_phaRP_* using In-Fusion HD Cloning Kit (Takara Bio), resulting in pWL102-P*_phaRP_*-*phaEC(X)* (*X*: abbreviated names of the haloarchaeal strain; *Hhi*, *H. hispanica*; *Hje*, *H. jeotgali*; *Hme*, *H. mediterranei*; *Hru*, *H. rubrum*; *Noc*, *N. occultus*). The sequence of pWL102-P*_phaRP_* and pWL102-P*_phaRP_*-*phaEC(X)* were confirmed by sequencing service (Fasmac, Kanagawa, Japan) with appropriate primers. These plasmids were introduced into the DEC strain using a polyethylene glycol-mediated transformation method (Cline et al., 1989), resulting in DEC*-phaEC(X)* strains. Transformants containing pWL102-P*_phaRP_* or pWL102-P*_phaRP_*-*phaEC(Hme)* in the DEC strain were used as negative (DEC-null) or positive controls, respectively. These transformants were confirmed to contain the desired plasmid using head and tail primer that specifically bind to the front and back of *Xba*I site on pWL102 by colony PCR with KOD FX DNA polymerase (Toyobo, Osaka, Japan).

### 2.3 Culture media compositions

All *E. coli* strains were cultured in Luria-Bertani medium containing 10 g/L NaCl, 10 g/L tryptone, and 5 g/L yeast extract. The 257 medium containing 156 g/L NaCl, 20 g/L MgSO_4_·7H_2_O, 13 g/L MgCl_2_·6H_2_O, 5 g/L yeast extract, 4 g/L KCl, 1 g/L glucose, 1 g/L CaCl_2_·2H_2_O, 0.5 g/L KBr, and 0.2 g/L NaHCO_3_ (pH 7.0) was used for transformation and pre-culture of derivative strains of *H. mediterranei*. The following media were used for the main culture. Modified basal synthetic (MBS) medium contained 194 g/L NaCl, 24 g/L MgSO_4_·7H_2_O, 16 g/L MgCl_2_·6H_2_O, 5 g/L yeast extract, 5 g/L KCl, 2 g/L NH_4_Cl, 0.5 g/L NaBr, 0.2 g/L NaHCO_3_, 37.5 mg/L KH_2_PO_4_, 5 mg/L FeCl_3_·6H_2_O (pH 7.2). The MBS medium was optimized for stable PHA production by *H. mediterranei* (Lillo and Rodriguez-Valera, 1990; Ino et al., 2020; Sato et al., 2021). The 1380 medium contained 200 g/L NaCl, 50 g/L MgSO_4_·7H_2_O, 2 g/L KCl, 1.8 g/L glucose, 1 g/L Trisodium citrate, 1 g/L NH_4_Cl, 1 g/L monosodium glutamate, 0.3 g/L K_2_HPO_4_, 0.1 g/L yeast extract, 0.1 g/L casamino acid, and 2 mL 500 × Trace element solution (12.8 g/L nitrilotriacetic acid, 1.35 g/L FeCl_3_·6H_2_O, 1 g/L NaCl, 0.12 g/L NiCl_2_·6H_2_O, 0.1 g/L MnCl_2_·4H_2_O, 0.1 g/L CaCl_2_·2H_2_O, 0.1 g/L ZnCl_2_, 25 mg/L CuCl_2_·2H_2_O, 24 mg/L CoCl_2_·6H_2_O, 24 mg/L Na_2_MoO_4_·2H_2_O, and 10 mg/L H_3_BO_3_) (pH7.0). The 1380P medium comprised the 1380 medium and 15 g/L piperazine-*N, N′*-bis(2-ethanesulfonic acid) (PIPES). The 1380PP medium comprised 1380P media and 0.5 g/L sodium propionate. The series of 1380 media were used to produce high-molecular-weight PHA (Sato et al., 2021). Propionate was added for increasing 3HV compositions in PHA (Fu et al., 2014). The 1337 medium contained 200 g/L NaCl, 20 g/L MgCl_2_·6H_2_O, 12.1 g/L Tris(hydroxymethyl)aminomethane, 5 g/L yeast extract, 5 g/L casamino acids, 2 g/L KCl, 0.2 g/L CaCl_2_·2H_2_O (pH 7.4). The 1338 medium contained 132 g/L NaCl, 3 g/L peptone, 1 g/L yeast extract, 1 g/L casamino acids, 1 g/L glucose, 0.6 g/L K_2_HPO_4_, 0.05 g/L sodium pyruvate (pH 7.1). The main culture media were supplemented with 5 g/L glucose as a substrate for PHBV production. The 257, 1338, and 1380 media were prepared according to the medium recipes released by the NBRC.

### 2.4 Culture conditions for DEC-null and DEC-*phaEC(X)* strains

For pre-culture, the DEC-null and DEC*-phaEC(X)* strains were inoculated in 5 mL of the 257 medium in test-tubes and were incubated at 37 °C with agitation at 200 rpm for 72 h. To evaluate PHA productivities, 1% volume precultures were transferred into 50 mL of MBS, 1380P, and 1380PP media in 300-mL Erlenmeyer flasks and cultured at 37 °C with agitation at 200 rpm for 72, 48, and 48 h, respectively. The cultivations were performed in triplicated. The difference in cultivation time for each medium came from the difference in the time taken for the wild-type strain of *H. mediterranei* to completely consume glucose added in each media (data not shown). A total of 0.5 mL of the culture broth was collected every 24 h to measure cell growth. Cell growth was monitored by measuring optical density at 600 nm (OD_600_) with a spectrophotometer V-630 (JASCO, Tokyo, Japan).

### 2.5 Quantification of composition and molecular weight of intracellular PHA

All procedures followed those described in our previous studies (Ino et al., 2020; Sato et al., 2021). To measure the amount of total PHA and the 3HV composition, cells in 2 mL of culture broth were harvested by centrifugation, washed with NaCl solution, and dried under reduced pressure. The pellets were subjected to methanolysis to form methyl esters from PHA and were used to determine the monomer composition in PHA by gas chromatography/mass spectrometry comprised of a 6890N and 5973 (Agilent Technology, Santa Clara, CA, USA) equipped with a TC-WAX column (GL Science Inc., Tokyo, Japan).

To measure the molecular weights, the cells in 2 mL of culture broth were harvested by centrifugation, washed with Milli-Q water, and dried under reduced pressure. The pellets were resuspended in chloroform overnight and subjected by size-exclusion chromatography using an HLC-8320GPC system (Tosoh Corporation, Tokyo, Japan) equipped with two TSKgel Super HZM-H columns (Tosoh). The column temperature was set to 40 °C, and chloroform was used as the eluent with a flow rate of 0.6 mL/min. A calibration curve was constructed for the quantification of standard polystyrene kit PStQuick (Tosoh). The number-average molecular weight (*M*_n_) and *M*_w_ were calculated by a software EcoSec (Tosoh).

### 2.6 Quantification of extracellular glucose concentration

To measure the glucose concentration, 0.5 mL of culture broth was centrifuged and filtered. The filtrates were subjected to high-performance liquid chromatography Nexera (Shimadzu, Kyoto, Japan) equipped with a KS-802 column (Showa Denko, Tokyo, Japan) and a refractive index detector RID-20A. The column temperature was set to 60 °C, and deionized water was used as the mobile phase with a flow rate of 1.0 mL/min. A calibration curve was constructed for the quantification of d-glucose. Biomass formation was calculated by subtracting the PHA from the dry cell weight (DCW).

### 2.7 Correlation analysis

To evaluate the relationship between each parameter for growth and PHA profiles in the DEC-*phaEC(X)* strains, Pearson’s correlation analysis was performed using R (version 4.4.2, *cor.test()* function) under the assumption that each variable was continuous and normally distributed. The following formula was used to estimate the correlation:


$$r=\frac{\sum (X_{i}-\bar{X})(Y_{i}-\bar{Y})}{\sqrt{\sum {\left(X_{i}-\bar{X}\right)}^{2}\cdot \sum {\left(Y_{i}-\bar{Y}\right)}^{2}}}$$

where $X_{i}$ and $Y_{i}$ represent the observed values of parameters *X* and *Y* for the i-th sample, and $\bar{X}$ and $\bar{Y}$ denote their respective sample means. The null hypothesis was that ρ=0 . Degrees of freedom were calculated as 3 , and statistical significance was set at *p* < 0.05.

### 2.8 Culture conditions for wild-type strains of *H. mediterranei*, *H. jeotgali*, and *H. rubrum*

The wild-type strain of *H. mediterranei* was inoculated into 5 mL of 257 medium in a test tube as a pre-culture and incubated with agitation at 200 rpm. For main culture, the pre-culture was transferred at 1% (v/v) into 50 mL of fresh 257 medium in a 300-mL Erlenmeyer flask and cultivated at 37 °C with agitation at 200 rpm for 72 h. The wild-type strain of *H. jeotgali* was inoculated into 5 mL of 1337 medium in a test tube as a pre-culture and incubated with agitation at 200 rpm. For main culture, the pre-culture was transferred at 1% (v/v) into 500 mL of fresh 1337 medium in a 2,000-mL Erlenmeyer flask and cultivated at 37 °C with agitation at 200 rpm for 120 h. The wild-type strain of *H. rubrum* was inoculated into 5 mL of 1338 medium in a test tube as a pre-culture and incubated with agitation at 200 rpm. For main culture, the pre-culture was transferred at 1% (v/v) into 500 mL of fresh 1338 medium in a 2,000-mL Erlenmeyer flask and cultivated at 37 °C with agitation at 200 rpm for 120 h. PHA composition was quantified by gas chromatography in triplicate, and the molecular weight of PHA produced by *H. jeotgali* was determined in single replicate.

## 3 Results

### 3.1 Screening of haloarchaeal strains as source of *phaEC*

**Figure 1:**
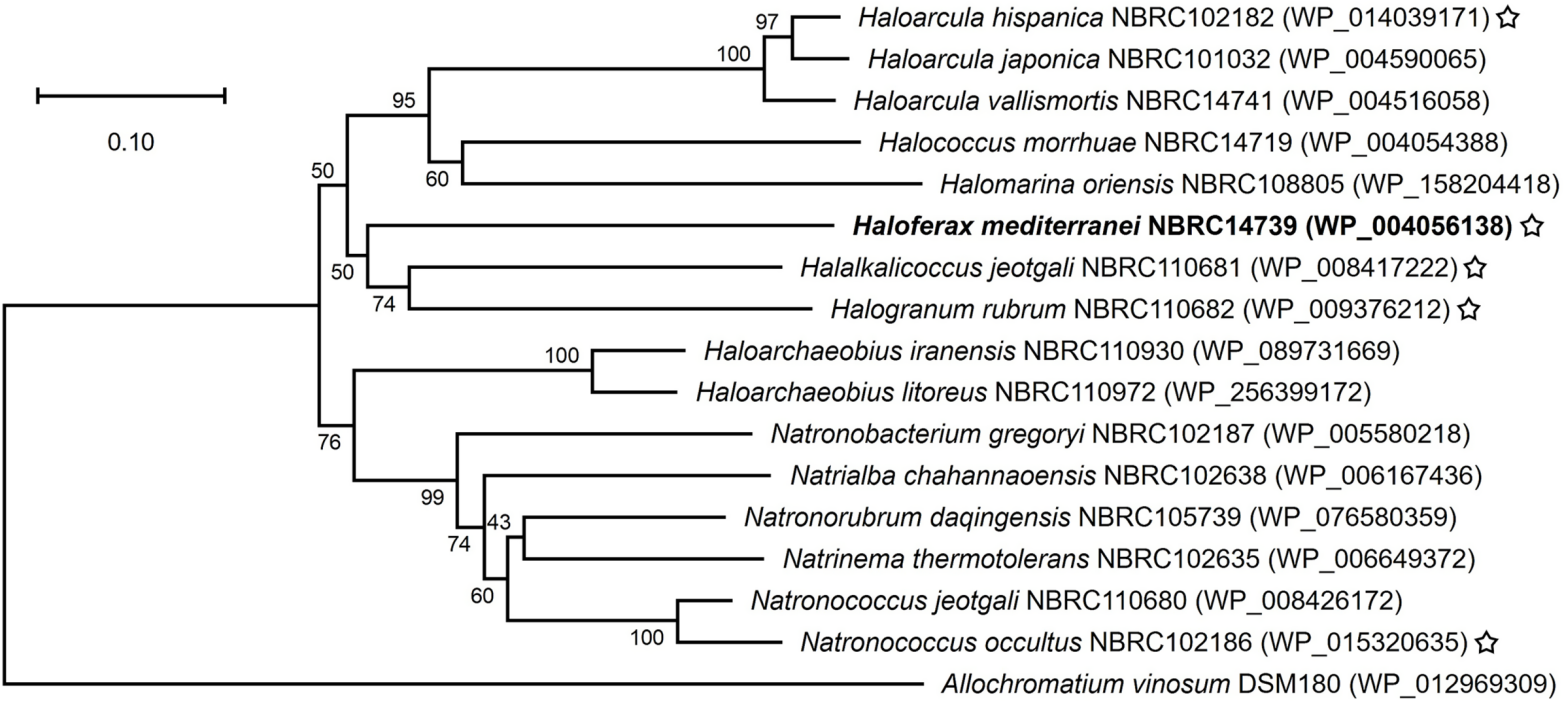
Phylogenetic tree based on amino acid sequence of PhaC from 16 haloarchaeal strains. The PhaC sequence from *A. vinosum* was used as an out-group. The GenBank accession numbers were given after the names of haloarchaeal strains. The numbers expressed as percentages next to the nodes indicated the bootstrap values. Bold: control, star; candidates.

Haloarchaeal PHA synthase is classified as a class III PHA synthase, which comprises the subunit PhaE and the catalytic subunit PhaC; its complex catalyzes the polymerization of 3-hydroxyacyl coenzyme A into PHA. All strains used in this study except *H. mediterranei* encoded a single *phaEC* gene cluster in their genomes (Supplementary Table S1). The genome of *H. mediterranei* encodes a set of *phaEC* and three *phaC*s. Transcriptional analysis revealed that only the *phaC* adjacent to *phaE* was expressed in PHA production conditions, suggesting that it is the main unit for PHA production (Han et al., 2010b). We compared sequences of PhaC adjacent to PhaE from 16 type strains among the haloarchaea. These 16 strains were selected because authenticated cultures were available from public culture collections and their complete genome sequences had been determined at the time of this study. These PhaCs comprised 449–525 amino acids with 50.1–57.5 kDa weights and were typical class III enzymes (Neoh et al., 2022). A phylogenetic tree was constructed based on the amino acid sequences of the PhaCs (Figure 1). Closely related genera and species were placed in close proximity, even in the PhaC sequence, such as the alignment based on the 16S rRNA sequence (Arahal et al., 1996). Previous studies have demonstrated that some haloarchaeal strains produce PHBV (Nicolaus et al., 1999; Lu et al., 2008). Particularly, PHBV produced by *H. mediterranei* exhibited ultrahigh molecular weights (Ino et al., 2020; Sato et al., 2021). On the other hand, little is known about the molecular weight of PHA produced by wild-type strains of other haloarchaea. PhaC sequences, which were expected to produce high-molecular-weight PHA, were selected based on the positions in the phylogenetic tree (Figure 1). A previous study reported that the wild-type *H. hispanica* produces PHBV (Han et al., 2009). PhaCs from *H. jeotgali* and *H. rubrum* were placed in the clade closest to *H. mediterranei*, indicating that they share a relatively close sequence homology. PhaC from *N. occultus* showed the greatest degree of change among the clusters, which was distinct from those of the aforementioned strains. Notably, a previous study demonstrated that *N. occultus* produces poly(3-hydroxybutyrate), but not PHBV (Han et al., 2010a). Thus, *phaEC*s from the five strains, including *H. mediterranei* as a control, were selected for further evaluation.

### 3.2 PHA profiles of the DEC-*phaEC(X)* strains in MBS medium

Since all PHA synthases from the haloarchaea strains selected in this study are classified as class III, not only the *phaC* gene but also *phaE* gene (which is immediately upstream of the *phaC* gene and functions as an indispensable subunit of PHA synthase) are necessary to produce PHA. The *phaEC* fragments from the five haloarchaeal strains were subcloned into pWL102-P*_phaRP_* and introduced into the DEC strain, resulting in the DEC-*phaEC(X)* strains. These strains were cultured in the MBS medium. The DEC-null possessing empty vector pWL102-P*_phaRP_* and DEC-*phaEC(Hme)* strains were used as negative and positive controls, respectively.

**Figure 2:**
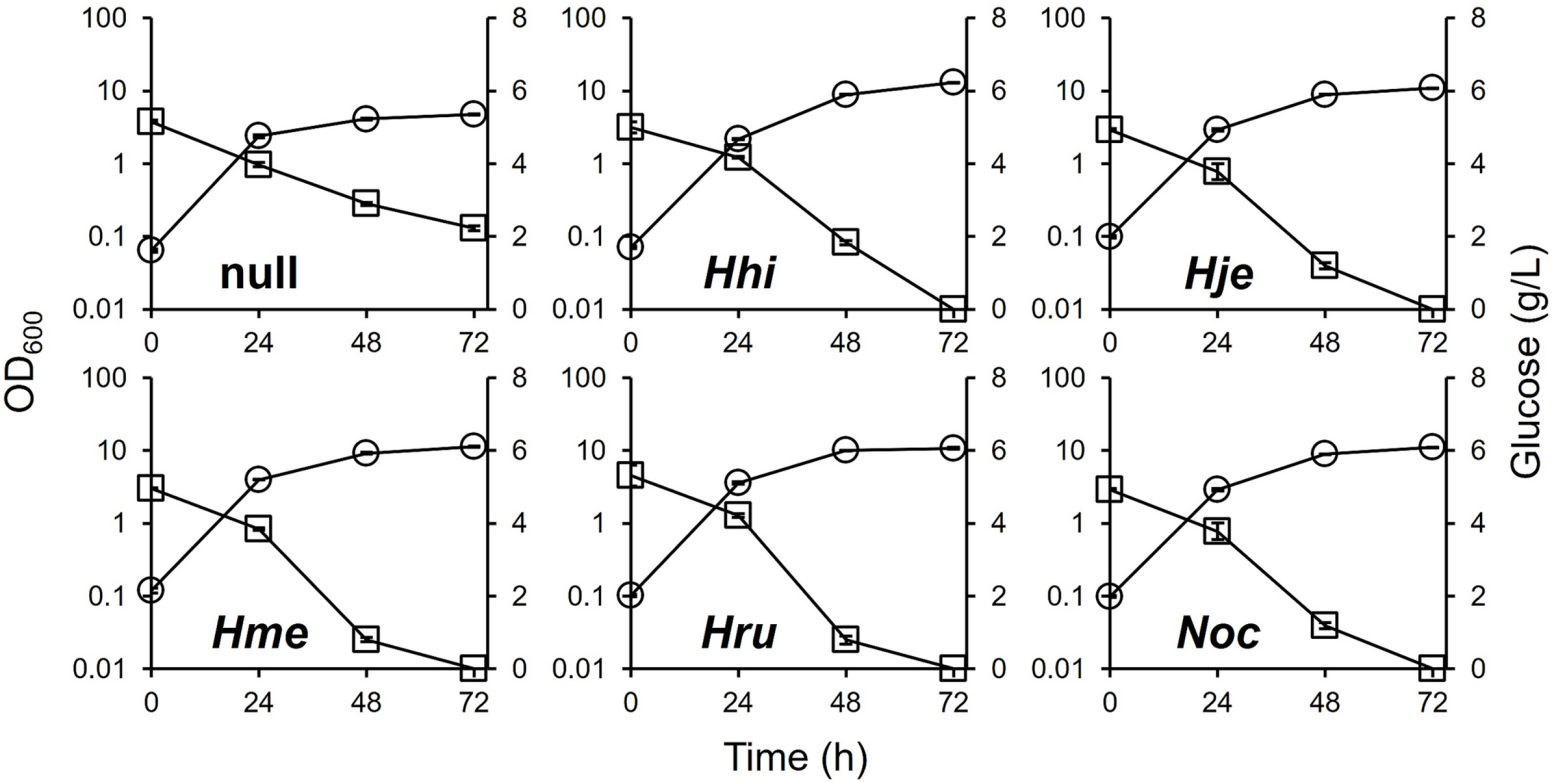
Culture profiles of the DEC-null and DEC-*phaEC(X)* strains in MBS medium. Circle; OD_600_, square; glucose. Data shown were mean ± SD (*N* = 3).

The culture profiles are shown in Figure 2. All DEC-*phaEC(X)* strains grew well (OD_600_ over 10) and showed efficient glucose consumption (residual glucose: approximately 0 g/L). In the DEC-null strain, which was unable to produce PHA due to the deletion of *phaEC*, the cell density was low. This may be due in part to the absence of the cell-volume increase typically associated with PHA accumulation; however, we cannot exclude the possibility that the strain also had a reduced cell number, as cell size and cell number were not directly examined in this study. While 56% (=2.9/5.2) of the initially added glucose remained at the end of the culture in the DEC-null strain, the DEC-*phaEC(Hme)* strain consumed the glucose completely within 72 h. This implied that PHA production directly contributed to glucose consumption.

To evaluate the effects of heterologous *phaEC* expression on PHA production, PHA in cells at the end of the culture was extracted and analyzed for composition and molecular weight (Table 2). All DEC-*phaEC(X)* strains produced PHBV and showed comparable levels of glucose consumption (4.9–5.3 g/L), DCW (3.5–4.0 g/L), PHBV production (1.4–1.7 g/L), and 3HV compositions (8–9 mol%). These strains also showed higher biomass formation than the DEC-null strain. The DEC-null strain showed slow glucose consumption, resulting in reduced biomass formation. These results suggest that PHA production positively affects growth. The molecular weights were distributed in the ranges of (0.5 ± 0.1)–(1.8 ± 0.1) × 10^6^ g/mol for *M*_n_ and (0.97 ± 0.01)–(2.9 ± 0.1) × 10^6^ g/mol for *M*_w_. The DEC-*phaEC(Hje)* and DEC-*phaEC(Noc)* strains produced PHBVs with molecular weights equal to or greater than those produced by the DEC-*phaEC(Hme)* strain. The profiles of PHBV produced in DEC-*phaEC(Hme)*, except for the molecular weight, were comparable to those produced by the wild-type strain (Sato et al., 2021). Contrastingly, the *M*_n_ and *M*_w_ observed in the DEC-*phaEC(Hme)* strain were less than half those observed in the wild-type strain.

**Table 2:** Composition and molecular weight of PHA produced by the DEC-*phaEC(X)* strains in MBS, 1380P, and 1380PP medium.

Media or strains	Consumed glucose (g/L)	Consumed propionate* (g/L)	DCW (g/L)	PHA (g/L)	Biomass (g/L)	PHA (%)	3HV (mol%)	*M*_n_ (×10^6^ g/mol)	*M*_w_ (×10^6^ g/mol)
MBS									
DEC-null	2.9 ± 0.1	N.D.	1.50 ± 0.05	N.D.	1.50 ± 0.05	N.D.	N.D.	N.D.	N.D.
DEC-*phaEC(Hhi)*	5.0 ± 0.2	N.D.	3.6 ± 0.1	1.5 ± 0.1	2.1 ± 0.1	41 ± 1	7.6 ± 0.1	0.5 ± 0.1	1.37 ± 0.01
DEC-*phaEC(Hje)*	4.93 ± 0.03	N.D.	3.5 ± 0.1	1.72 ± 0.04	1.75 ± 0.04	49 ± 1	8.4 ± 0.1	1.35 ± 0.05	2.26 ± 0.03
DEC-*phaEC(Hme)*	4.96 ± 0.03	N.D.	3.6 ± 0.1	1.69 ± 0.04	1.90 ± 0.04	47 ± 1	8.8 ± 0.1	1.21 ± 0.04	2.2 ± 0.1
DEC-*phaEC(Hru)*	5.3 ± 0.3	N.D.	3.98 ± 0.03	1.45 ± 0.04	2.53 ± 0.04	36 ± 1	9.3 ± 0.1	0.61 ± 0.01	0.97 ± 0.01
DEC-*phaEC(Noc)*	5.0 ± 0.1	N.D.	4.0 ± 0.1	1.6 ± 0.1	2.4 ± 0.1	39 ± 1	7.9 ± 0.1	1.8 ± 0.1	2.9 ± 0.1
1380P									
DEC-null	5.0 ± 0.1	N.D.	2.0 ± 0.1	N.D.	2.0 ± 0.1	N.D.	N.D.	N.D.	N.D.
DEC-*phaEC(Hhi)*	6.7 ± 0.1	N.D.	3.08 ± 0.04	0.988 ± 0.004	2.09 ± 0.04	32.1 ± 0.3	10.8 ± 0.1	1.0 ± 0.1	2.1 ± 0.1
DEC-*phaEC(Hje)*	6.9 ± 0.2	N.D.	3.7 ± 0.1	1.16 ± 0.01	2.5 ± 0.1	31.7 ± 0.4	12.1 ± 0.1	1.7 ± 0.1	3.16 ± 0.03
DEC-*phaEC(Hme)*	6.91 ± 0.01	N.D.	3.6 ± 0.1	1.18 ± 0.01	2.4 ± 0.1	33 ± 1	10.75 ± 0.03	1.02 ± 0.05	1.88 ± 0.02
DEC-*phaEC(Hru)*	6.4 ± 0.2	N.D.	2.98 ± 0.02	0.84 ± 0.02	2.13 ± 0.04	28 ± 1	11.9 ± 0.1	1.03 ± 0.05	1.59 ± 0.02
DEC-*phaEC(Noc)*	6.8 ± 0.2	N.D.	3.3 ± 0.1	0.95 ± 0.05	2.3 ± 0.1	29 ± 1	12.3 ± 0.2	1.7 ± 0.1	2.92 ± 0.03
1380PP									
DEC-null	5.50 ± 0.02	0.396 ± 0.005	2.2 ± 0.1	N.D.	2.2 ± 0.1	N.D.	N.D.	N.D.	N.D.
DEC-*phaEC(Hhi)*	5.8 ± 0.1	0.39 ± 0.01	3.1 ± 0.1	1.08 ± 0.04	2.1 ± 0.1	34 ± 1	27.4 ± 0.3	0.82 ± 0.03	1.8 ± 0.1
DEC-*phaEC(Hje)*	6.3 ± 0.3	0.38 ± 0.01	3.2 ± 0.1	1.1 ± 0.1	2.15 ± 0.01	33 ± 2	25 ± 1	1.8 ± 0.1	3.33 ± 0.04
DEC-*phaEC(Hme)*	6.5 ± 0.1	0.40 ± 0.01	3.4 ± 0.2	1.3 ± 0.1	2.2 ± 0.1	37 ± 1	23.7 ± 0.5	1.0 ± 0.1	1.7 ± 0.1
DEC-*phaEC(Hru)*	5.4 ± 0.3	0.33 ± 0.02	2.8 ± 0.1	0.7 ± 0.1	2.13 ± 0.04	25 ± 1	31 ± 2	0.67 ± 0.03	1.08 ± 0.02
DEC-*phaEC(Noc)*	5.9 ± 0.1	0.40 ± 0.01	3.19 ± 0.01	1.08 ± 0.01	2.10 ± 0.02	34.0 ± 0.4	26.1 ± 0.4	1.2 ± 0.1	2.0 ± 0.1

“N.D.” indicates “not detected”.

Sodium propionate was recalculated to propionic acid.

### 3.3 PHA profiles of the DEC-*phaEC(X)* strains in 1380P medium

We previously reported that although the wild-type strain of *H mediterranei* produced UHMW-PHBV (3HV, 26 mol%; *M*_w_, 5.8 × 10^6^ g/mol), PHBV productivity and biomass formation were lower in the 1380 medium, which contains limited organic nutrients, than in the MBS medium (Sato et al., 2021). The decrease in these parameters in the 1380 medium may be due to a decrease in glucose consumption caused by a change in pH during cultivation (data not shown). Since decrease in glucose consumption leads to an insufficient supply of PHA precursors, such as 3HB and 3HV, decrease in glucose consumption should be suppressed when evaluating the functionalities of *phaEC*. Hence, we combined PIPES, a buffering agent used in other haloarchaeal culture media (Zhao et al., 2013), with 1380 (1380P) medium to promote glucose consumption by preventing pH changes. The wild-type *H. mediterranei* strain was cultured in 1380 and 1380P media for 72 h (Supplementary Table S3). As expected, the DCW, PHA productivity, and biomass formation increased with increasing glucose consumption. Additionally, the *M*_n_ and *M*_w_ values of the PHBVs produced in both media were comparable, suggesting that the molecular weight of PHBV was maintained regardless of the presence or absence of PIPES. Therefore, we used 1380P medium to cultivate the DEC-*phaEC(X)* strains.

**Figure 3:**
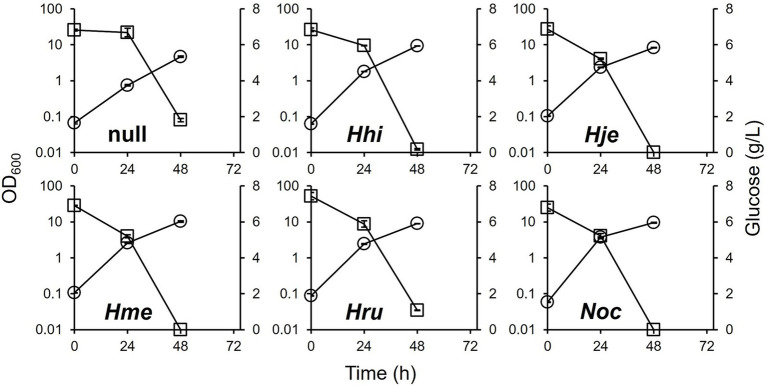
Culture profiles of the DEC-null and DEC-*phaEC(X)* strains in 1380P medium. Circle; OD_600_, square; glucose. Data shown were mean ± SD (*N* = 3).

The culture profiles of the DEC-*phaEC(X)* strains in 1380P medium are shown in Figure 3. Like in the MBS medium, all DEC-*phaEC(X)* strains grew well (OD_600_: approximately 10). While the DEC-*phaEC(Hru)* strain was unable to consume glucose fully, the other strains consumed almost all the glucose. This indicated that the catalytic efficiency of PhaECs for *H. rubrum* may be low *in vivo* because there were no differences other than *phaEC* among the DEC-*phaEC(X)* strains.

The PHA profiles produced in 1380P medium by all the strains are summarized in Table 2. All DEC-*phaEC(X)* strains produced PHBV and showed comparable levels of glucose consumption (6.7–6.9 g/L), DCW (3.1–3.7 g/L), PHBV production (1.0–1.2 g/L), biomass formation (2.1–2.5 g/L), and 3HV composition (11–12 mol%) except the DEC-*phaEC(Hru)* strain. These parameters were slightly lower in the DEC-*phaEC(Hru)* strain than in the other strains. The differences in biomass formation between the DEC-null strain and the other strains were less clear in the 1380P medium than in the MBS medium. The 3HV composition in all strains was slightly higher than that in the MBS medium. In the 1380P medium, unlike in the MBS medium, PHBV produced by the DEC-*phaEC(Hje)* and the DEC-*phaEC(Noc)* strains had higher *M*_n_ and *M*_w_ than that produced by the DEC-*phaEC(Hme)* strain. Particularly, the DEC-*phaEC(Hje)* strain produced PHBV with the highest molecular weight measured.

### 3.4 PHA profiles of the DEC-*phaEC(X)* strains in 1380PP medium

In the 1380P medium, glucose consumption was promoted by mitigating pH change in the presence of PIPES; however, no significant difference observed in the 3HV composition in all strains. To promote the introduction of 3HV into PHBV, propionate, a precursor of 3HV, was added to the 1380P medium, resulting in the 1380PP medium.

**Figure 4:**
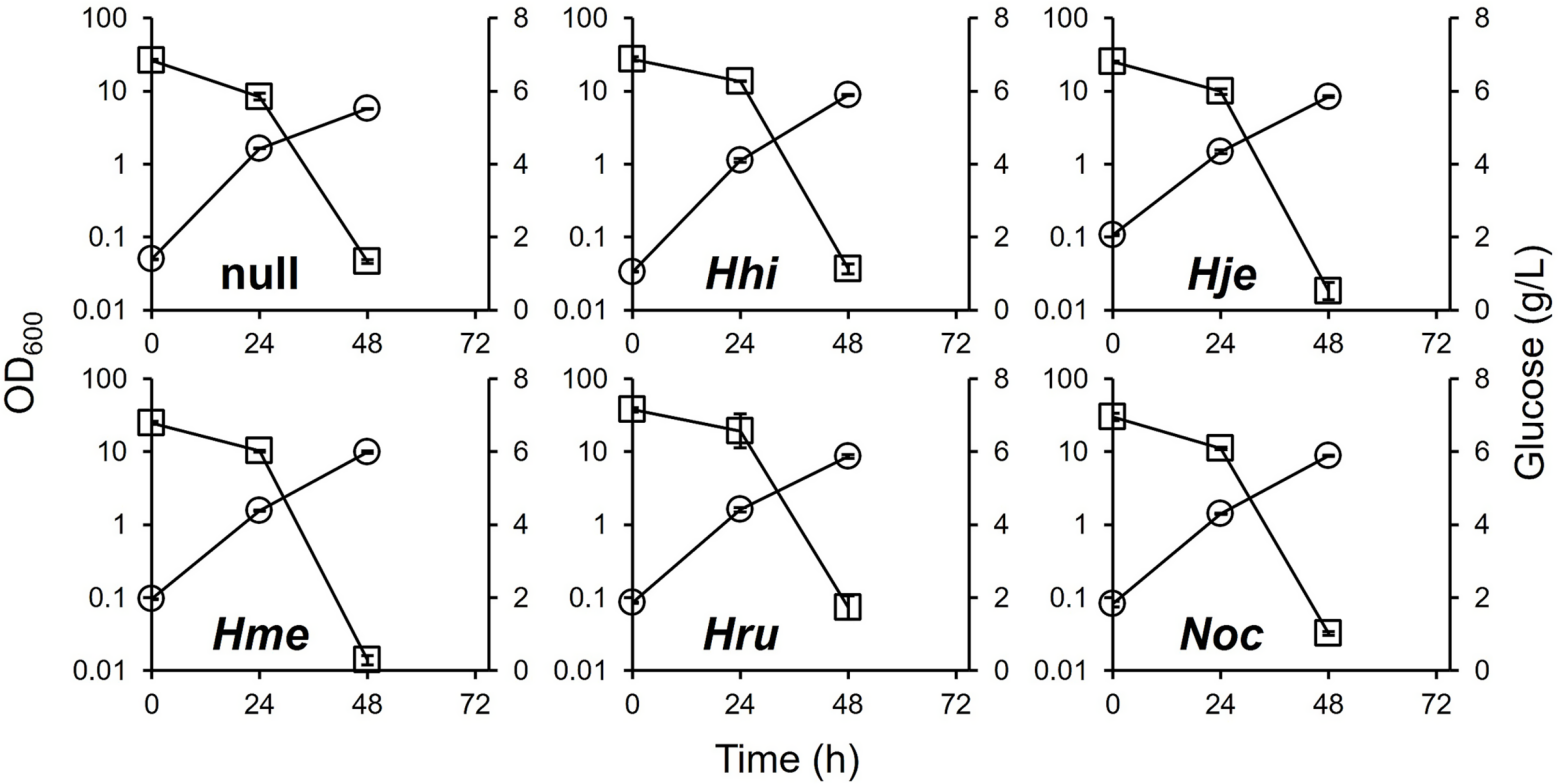
Culture profiles of the DEC-null and DEC-*phaEC(X)* strains in 1380PP medium. Circle; OD_600_, square; glucose. Data shown were mean ± SD (*N* = 3).

The culture profiles of all strains in the 1380PP medium are shown in Figure 4. All strains, except the DEC-*phaEC(Hme)* and DEC-*phaEC(Hje)* strains, retained more than 1 g/L glucose in their cultures. Glucose uptake by all strains, except DEC-null strain, was affected by stricter restrictions in the 1380PP medium, accompanied by a slowdown in growth curve compared with that in the 1380P medium. These results suggest that growth and glucose consumption were suppressed by propionate toxicity. The added propionate was fully consumed in all strains within 48 h. Although the DEC-*phaEC(Hme)* and DEC-*phaEC(Hje)* strains showed slower growth and glucose consumption due to the presence of propionate, the final OD_600_ and residual glucose concentration were comparable to those in the 1380P medium.

The PHA profiles produced in 1380PP medium by all the strains are summarized in Table 2. In the 1380PP medium, DCW and PHBV production in the DEC-*phaEC(Hru)* strain was lower than that in the DEC-*phaEC(Hme)* strain. Biomass formation was comparable across all the strains. As expected, the 3HV compositions in all DEC-*phaEC(X)* strains were higher and more variable (24–31 mol%) than those observed in MBS and 1380P media. The *M*_n_ and *M*_w_ in all DEC-*phaEC(X)* strains, except for the DEC-*phaEC(Hje)* strain, decreased compared to the values observed in 1380P medium. These results suggested that PhaEC from *H. jeotgali* has great potential for adding copolymer components and polymerizing high-molecular-weight-PHA, comparable to or greater than those of *H. mediterranei*.

**Figure 5:**
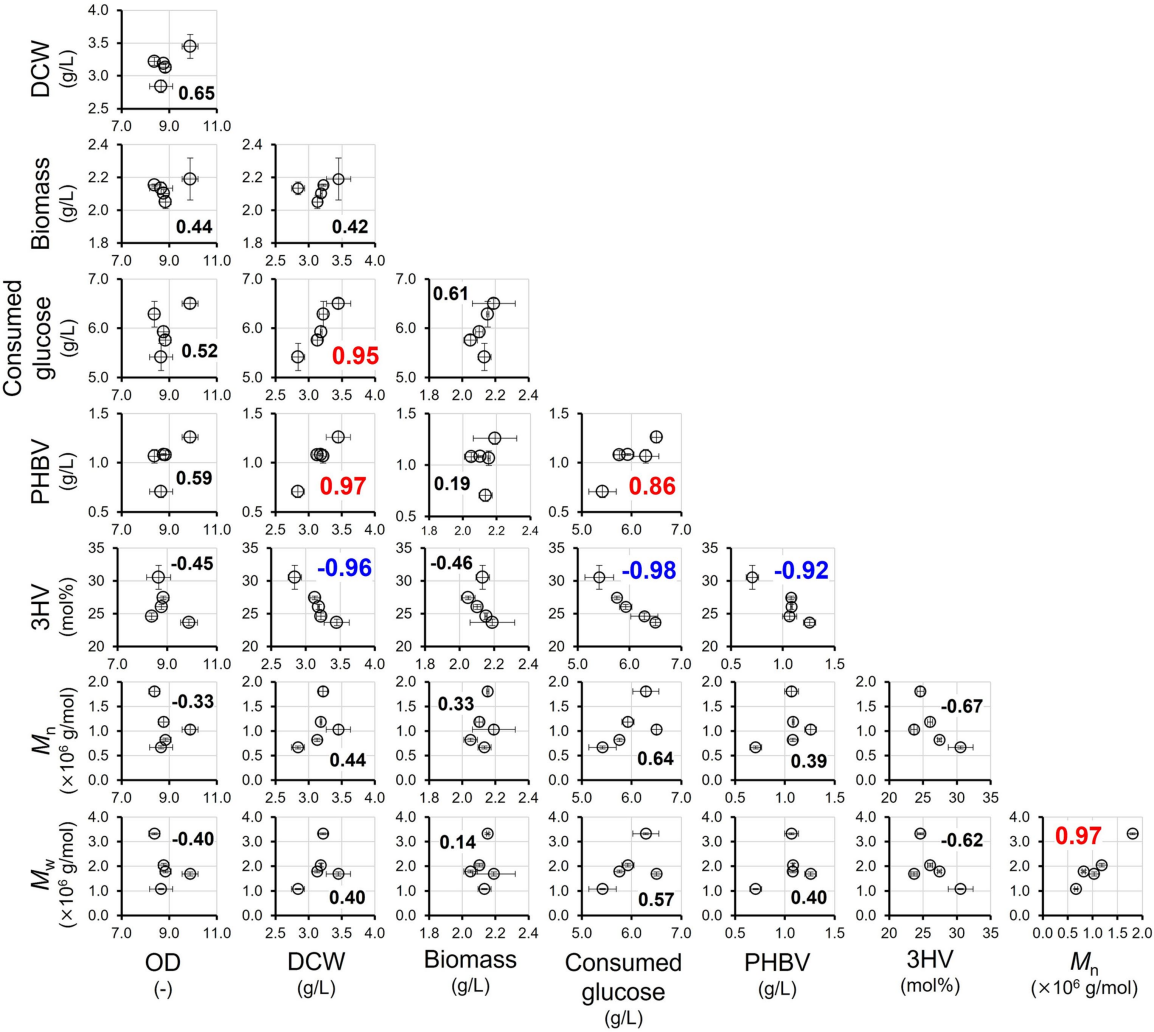
Correlation analysis using the data obtained from profiles of culture and PHA by the DEC-*phaEC(X)* strains. Bold: Pearson’s correlation coefficients, red; coefficients over 0.7, blue; coefficients negative under −0.7.

Correlation analysis using the profiles of culture and PHA in DEC-*phaEC(X)* is summarized in Figure 5. All reported correlations were calculated using Pearson’s correlation test. Statistical significance was assessed with a two-tailed test, and all correlations mentioned as “very strong” were significant at *p* < 0.05. Detailed correlation coefficients and *p*-value are provided in Supplemental Table S6. There were very strong positive correlations (> 0.7) between the following: glucose consumption vs. DCW and PHBV production; PHBV production vs. DCW; PHBV production vs. glucose consumption; and *M*_n_ vs. *M*_w_. The consumption of glucose (the main substrate) is highly correlated with DCWs and intracellular PHBVs. The *M*_n_ and *M*_w_ were also highly correlated. Contrastingly, there were very strong negative correlations (< −0.7) between 3HV composition vs. DCW, glucose consumption, and PHBV production. These results suggest that the introduction of 3HV into PHA interferes with the fundamental metabolism of PHA-producing cells. Strong negative correlations were observed between the 3HV composition and *M*_n_ and *M*_w_. This suggests that it is difficult to achieve an increase in both the 3HV composition and molecular weight. Hence, *phaEC* from *H. mediterranei* and *H. jeotgali*, which can increase the 3HV composition while maintaining the molecular weight by adding propionate, is useful for producing high-molecular-weight PHA with a high 3HV composition.

### 3.5 PHA production by the native strains of *H. jeotgali* NBRC110681 and *H. rubrum* NBRC110682

PHAs produced by the wild-type *H. jeotgali* NBRC110681 and *H. rubrum* NBRC110682 (whose PhaCs showed high homology with that of *H. mediterranei* NBRC14739) were measured. PHAs from *H. mediterranei* NBRC14739 cells were cultured in 257 media for 72 h as a control, *H. jeotgali* NBRC110681 cell were cultured in 1337 medium for 120 h, and *H. rubrum* NBRC110682 cells were cultured in 1338 medium for 120 h. The medium used for cultivating each strain was recommended by the NBRC. The PHA compositions and molecular weights are listed in Supplementary Table S4. PHA was not detected in the sample from *H. rubrum* NBRC110682. Contrastingly, *H. jeotgali* produced PHBV with 3.4-fold (=24/7) higher 3HV content than *H. mediterranei*, whereas its productivity was only approximately 0.3% (=8.6/2,375) of that of *H. mediterranei*. The elution profile of PHBV produced by *H. jeotgali* measured using a size-exclusion chromatography had bimodal peaks around 7.2 and 8.7 min for unknown reasons (Supplementary Figure S1). Its *M*_w_ was 2.8 × 10^6^ g/mol, comparable to that of PHBV produced by *H. mediterranei* (3.6 × 10^6^ g/mol). These results demonstrated that even the native strain of *H. jeotgali* NBRC110681 can produce PHBV with high molecular weight.

## 4 Discussion

In this study, we investigated the characteristics of *phaEC*s from haloarchaea under uniform conditions using a heterologous expression system with a PHA-negative mutant strain of *H. mediterranei*. Using five *phaEC*s selected based on the PhaC sequence alignment (Figure 1), we cultivated recombinant strains harboring each *phaEC* to investigate PHA production using three types of culture media. The strain harboring *phaEC* from *H. mediterranei* alongside the other four recombinant strains, produced PHBVs (see Table 2). This indicates that the expression system worked as designed, and *phaEC*s from haloarchaea functioned as PHA synthase genes in *H. mediterranei* cells. The productivity and structure of PHBVs varied depending on the *phaEC* introduced. Particularly, a strain possessing *phaEC* from *H. jeotgali* produced PHBV with *M*_w_ > 3.0 × 10^6^ g/mol in the medium containing low organic nutrients. Despite the negative correlation between 3HV composition vs. basal metabolic capacity and molecular weight (Figure 5), this strain achieved high 3HV composition and molecular weight. The native *H. jeotgali* NBRC110681 strain also produced PHBV with a high molecular weight (Supplementary Figure S1, Supplementary Table S3), suggesting that *phaEC* from *H. jeotgali* has the potential to produce PHBV with a high molecular weight, similar to *H. mediterranei*.

The previous studies on PHA production by the native haloarchaea were summarized in Supplementary Table S1. *H. mediterranei* (Lu et al., 2008) and *H. hispanica* (Han et al., 2009), and *N. occultus* (Han et al., 2010a) produced PHBV and PHB, respectively. *H. jeotgali* and *H. rubrum* have not been reported as PHA producers. On the other hand, all DEC-*phaEC(X)* strains produced PHBV, suggesting at least the haloarchaeal *phaEC*s tested can utilize both 3HB and 3HV as substrates. Wild-type *H. hispanica* showed greater cell growth with lower PHBV production than DEC-*phaEC(Hhi)*. The 3HV composition of the PHBV produced by *H. hispanica* was lower than that of the PHBV produced by the DEC-*phaEC(Hhi)* strain. Wild-type *N. occultus* did not produce PHBV despite the presence of *phaEC* in its genome, whereas the DEC-*phaEC(Noc)* strain produced PHBV in this study. These differences may be due to the metabolic capabilities of the host microbes, such as substrate consumption and monomer supply. Host-derived effects should be excluded when evaluating the functionality of heterologous *phaEC*s *in vivo*. A previous study succeeded in demonstrating the differences in PHB produced by heterologous PhaC in uniform conditions using *E. coli* as a host (Agus et al., 2006). Therefore, in the present study, we used the DEC strain as a host to eliminate genotypic differences other than those of *phaEC* as much as possible, allowing us to evaluate differences in the functionality of *phaEC*s.

The molecular weight of the PHBVs produced by DEC-*phaEC(Hme)* in all media tested was smaller than that of the PHBVs produced by the native strain of *H. mediterranei*. For example, in the MBS medium, the *M*_n_ of PHBVs produced by the DEC-*phaEC(Hme)* strain and native strain of *H. mediterranei* were (1.21 ± 0.04) × 10^6^ g/mol (Table 2) (0.8 ± 0.1) × 10^6^ g/mol (Sato et al., 2021), respectively. These strains differ in their *phaEC* expression systems. Expression of *phaEC* in the native *H. mediterranei* is regulated by the *phaEC* promoter (Lu et al., 2008), whereas that in DEC-*phaEC(Hme)* is regulated by a strong constitutive *phaRP* promoter (Cai et al., 2015), inferring larger amount of PhaEC in DEC-*phaEC(Hme)* than native strain. On the other hand, PHA productivity and biomass formation in both strains were comparable (Table 2), suggesting that the metabolic supply for PHBV and cell components was comparable. A previous study demonstrated that an increase in the supply of monomer components per unit amount of PhaC led to an increase in the molecular weight (Hiroe et al., 2013). Thus, the difference in the molecular weights of PHBV may result from differences in the expression levels of *phaEC* between the DEC-*phaEC(Hme)* strain and the native strain of *H. mediterranei*. Further studies measuring the number of PhaECs and monomer supply in each transformant will contribute to uncovering more detailed stoichiometries of molecular weight control.

In MBS and 1380P media, the amount and molecular weight of PHBV produced by all transformants varied, whereas the 3HV composition was constant (Table 2). This result suggests that the carbon source consumed by the DEC-*phaEC*(X) strains was always metabolized to 3HB and 3HV at a constant ratio, regardless of the *phaEC* introduced. However, the 3HV composition in PHBV produced in cells grown on 1380PP medium varied in the range of 24–31 mol% (Table 2). The amount of 3HB in PHBV produced by the DEC-*phaEC(Hru)* strain was lower than that produced by the other strains (Supplementary Table S5), resulting in the formation of a state with a high 3HV composition. These results suggest that the substrate specificity of PhaEC derived from *H. rubrum* may be different from those of PhaECs derived from the other strains. Furthermore, organic acids such as propionate are known to inhibit cell growth (Fu et al., 2014; Han et al., 2015), consistent with the slowing down of glucose consumption in some strains in the presence of propionate (Table 2). Therefore, in the transformants used in this study, propionate toxicity may have been avoided by introducing 3HV converted from propionate into PHBV. However, further analyses are required to prove this hypothesis.

In the present study, all phenotypic differences were triggered by *phaEC* introduced into the DEC strain. All DEC-*phaEC(X)* strains showed differences in the growth profiles, 3HV compositions, and molecular weights of the PHBV produced depending on the culture conditions. In the presence of propionate, differences in the growth profiles and 3HV composition among the DEC-*phaEC(X)* strains were particularly noticeable. These findings suggest that at least *phaEC* from the haloarchaeal strains used in this study had the potential to produce PHBVs with high molecular weight to a greater or lesser degree. However, differences in substrate specificity and molecular weight control mechanisms remain unclear. Conversely, previous studies have suggested that *phaEC*s with slightly different amino acid sequences are widely conserved in the genomes of various haloarchaea (Wang et al., 2019; Vuong et al., 2021; Rodríguez-Aristizabal et al., 2025). The structures of PHAs synthesized by these *phaEC*s are likely to be as diverse as the results of this study. Notably, we found that *phaEC*s from *H. jeotgali* and the wild-type strain were capable of producing high-molecular-weight PHBV similar to *phaEC* from *H. mediterranei*. This could facilitate not only the elucidation of mechanisms underlying high-molecular-weight PHBV production, but also the development of tailor-made PHBV production using biotechnological techniques, such as comparative analyses of the three-dimensional structure of PhaECs. Although notable challenges persist, the success of these improvements will open a new frontier for PHA production by microbes extending beyond haloarchaea.

Our results suggest that differences in PHBV molecular weight among transformants are not solely due to host metabolic capacity but are likely influenced by the expression level of *phaEC*. The DEC strains expressed *phaEC* under a strong constitutive promoter, which may have altered the stoichiometry between enzyme abundance and monomer supply, thereby affecting polymer chain elongation. While this indicates that *phaEC* expression level is a critical factor in controlling molecular weight, this conclusion is based on indirect evidence and requires further quantitative analysis of enzyme abundance and precursor flux. Within the scope of this study, we demonstrated functional diversity among haloarchaeal *phaEC* under uniform conditions, but the precise mechanisms of molecular weight regulation remain unresolved. Future work should integrate expression profiling and metabolic flux analysis to confirm the role of *phaEC* expression in PHA biosynthesis.

## Data Availability

The original contributions presented in the study are included in the article/supplementary material.
