# Supplementary figures and images for: Heterologous expression and characterization of polyhydroxyalkanoate synthase genes from haloarchaeal strains in Haloferax mediterranei
Source: Front Microbiol. 2026 Mar 18;17:1754904. doi: 10.3389/fmicb.2026.1754904 (PMC13038957; doi:10.3389/fmicb.2026.1754904)

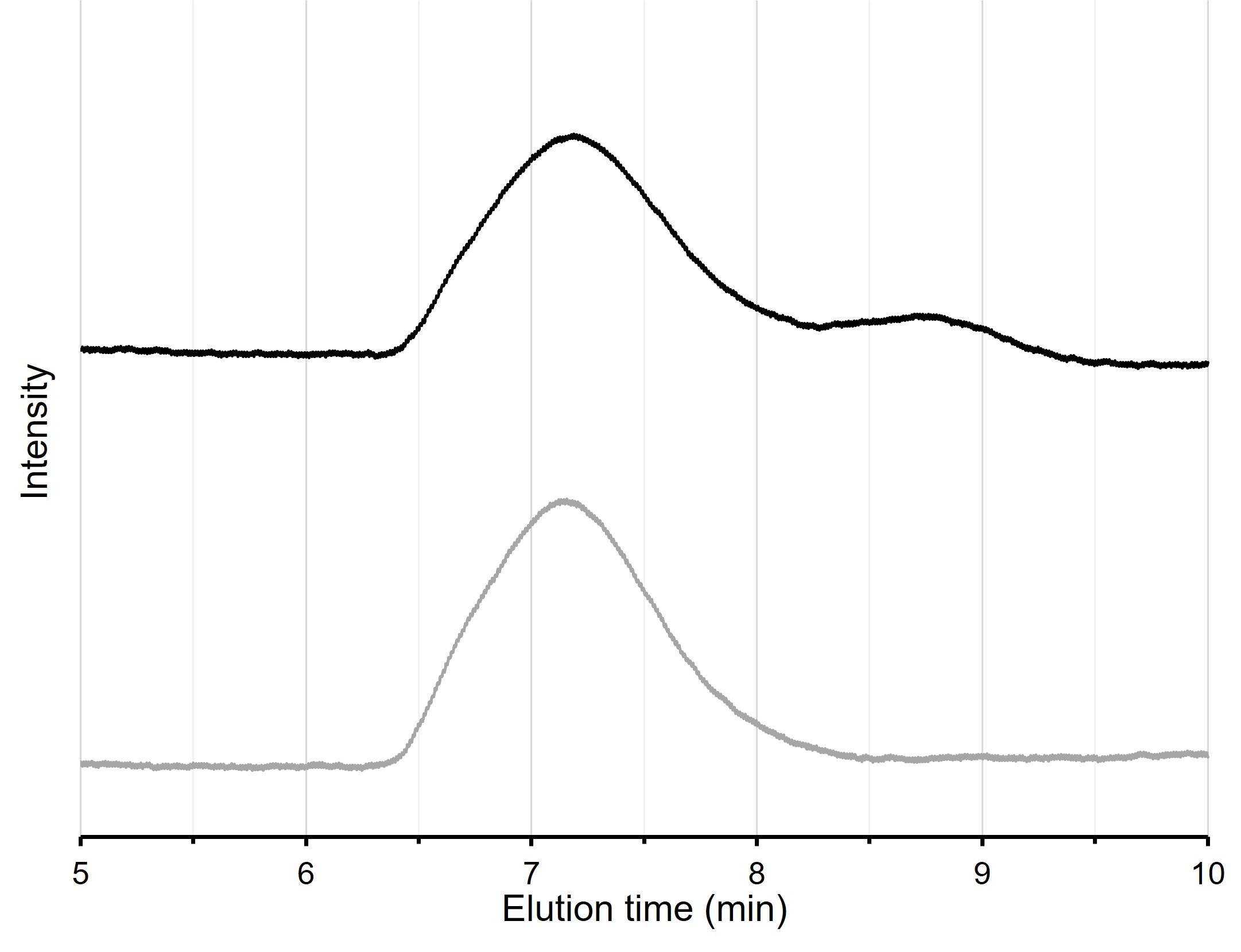

Supplement: Supplementary file 2 [file Image_1.jpeg]
